# An objective structured biostatistics examination: a pilot study based on computer-assisted evaluation for undergraduates

**DOI:** 10.3352/jeehp.2012.9.9

**Published:** 2012-07-17

**Authors:** Abdul Sattar Khan, Hamit Acemoglu, Zekeriya Akturk

**Affiliations:** 1 Family Medicine Department, Ataturk University-Erzurum, Turkey.; 2 Medical Education Department, Ataturk University-Erzurum, Turkey.

**Keywords:** Objective structured biostatistics examination, Assessment

## Abstract

We designed and evaluated an objective structured biostatistics examination (OSBE) on a trial basis to determine whether it was feasible for formative or summative assessment. At Ataturk University, we have a seminar system for curriculum for every cohort of all five years undergraduate education. Each seminar consists of an integrated system for different subjects, every year three to six seminars that meet for six to eight weeks, and at the end of each seminar term we conduct an examination as a formative assessment. In 2010, 201 students took the OSBE, and in 2011, 211 students took the same examination at the end of a seminar that had biostatistics as one module. The examination was conducted in four groups and we examined two groups together. Each group had to complete 5 stations in each row therefore we had two parallel lines with different instructions to be followed, thus we simultaneously examined 10 students in these two parallel lines. The students were invited after the examination to receive feedback from the examiners and provide their reflections. There was a significant (P=0.004) difference between male and female scores in the 2010 students, but no gender difference was found in 2011. The comparison among the parallel lines and among the four groups showed that two groups, A and B, did not show a significant difference (P>0.05) in either class. Nonetheless, among the four groups, there was a significant difference in both 2010 (P=0.001) and 2011 (P=0.001). The inter-rater reliability coefficient was 0.60. Overall, the students were satisfied with the testing method; however, they felt some stress. The overall experience of the OSBE was useful in terms of learning, as well as for assessment.

In medical science the most significant domains are the ability to think critically, to diagnose a case, and to manage it appropriately; thus it has been suggested to assess these skills step by step [1, 2]. However, perhaps public health requires a more rigorous problem solving approach to assess practical skills and biostatistics even further needs a comprehensive analytical approach [3]. The statistics in the biosciences is considered an essential component of the under- and postgraduate curriculum and the application of biostatistics needs a thorough understanding of the use of computer analytical software tools [4] too. In addition, today's new technologies play a role in transitioning a university from traditional to paperless sources of information, giving knowledge its new shape [5].

There are different levels of learning have been discovered so far, and according to these levels, the cognitive skills should result in behavioral changes; however, to measure these changes seems a difficult task [6]. One method of assessment for this area that is being increasingly used is the objective structured clinical examination (OSCE) in undergraduate and postgraduate examinations, and research has shown that it is an effective evaluation tool for assessing problem solving and practical skills [7-9]. Similarly, we designed and evaluated a computer-assisted objective structured biostatistics examination (OSBE) on a trial basis to determine whether it was feasible for formative or summative assessment.

Study design and procedure: This was a multi-method study including an exploratory and descriptive study design. The exploratory design mainly focused on measuring benefits that target students may gain from usage of computers to assist in improving computer and analytical skills while preparing and appearing in the OSBE. The descriptive design was mainly for gathering student feedback. The candidates had completed the scheduled mandatory computer skills training on SPSS software (SPSS Inc., Chicago, IL, USA) with the faculty during their biostatistics course. There were 05 stations in the OSBE, which comprised stations with a focus on different commands related to data entry and analysis (Table 1). The two phases (Year 2009, 2010) had different commands according to their learning objectives and course completion. Each station had three elements: one examiner, one candidate, and one computer. The SPSS ver. 18 was loaded onto all of the computers. The total number of students was 201 in the 2009-2010 examination and 211 in 2010-2011. All of the students were divided into 4 groups and were gathered in a large room before starting the examination. The examination was conducted in one large room and stations were positioned in two rows with different commands, so we simultaneously examined 10 students in these two parallel lines. The students had 2 minutes to complete each station. The total time for the assessment process was around 15 minutes for each student; however, the waiting time is around 5 minutes. No rest station was scheduled, and whole process was completed in half a day. The students were not allowed to meet their colleagues to prevent contamination. After compilation of the results, the students were invited to discuss the results and feedback was provided. In addition, we asked the students how they felt about the examination process.

Study setting: At Ataturk University, we have a seminar system for the curriculum for every cohort from the first year to fifth year. Each seminar consists of an integrated system of different subjects and every year has three to six seminars. Each seminar runs for six to eight weeks and at the end of each seminar, we conduct an examination as a formative assessment. The study took place in the Department of family medicine, Ataturk University during 2010-11. The examiners and candidates were given a briefing session before the OSBE, where the goals and objectives of the study were also explained, queries and concerns were addressed, and consent for participation was collected. The research committee at the university approved the study.

Instrument and data collection: A rating scale was developed, consisting of 5 items relevant to specific software handling, data entry, correct identification of data, and appropriate application of statistical tests. It was discussed with other senior faculty in order to check its face and content validity and was then applied in a real situation in order to observe for pre-testing. Input was also solicited from colleagues about whether they agreed with the items and rating scales or not.

Data analysis: All of the variables were examined for outliers and non-normal distributions. A two-way analysis of variance (TWANOVA) was used to determine any between group effects (groups and parallel groups), within-subject effects, and interactions between groups and parallel groups. Cronbach's alpha was computed for inter-rater reliability. Analyses were completed using SPSS ver. 18.0. Statistical significance for all analyses was set at P<0.05.

The results of the OSBE illustrate (Table 2) that in phase 1 (year one), 61% of the participants were males while in phase 2, 56.4% were males. The total mean scores for the males in phase 1 was 9.5±3.3, whereas for the females it was 10.9±3.4. In phase 2 (the second year), the total mean score for males was 3.4±1.3 and for females was 3.5±1.2. There is a significant (P=0.004) difference in the males and females of the phase 1 students; however, in phase 2, there is no significant difference in their scores. The comparison between the parallel groups and among the four groups shows that the two groups A and B do not have any significant difference (P>0.05) in either phase. However, among the four groups, there is a significant difference in phase 1 (P=0.001) and phase 2 (P=0.001). Inter-rater reliability was calculated around (Cronbach's alpha) 0.60. Overall, the students were satisfied; however, a majority (62%) were under stress and confused because of the first experience. Almost 18% identified that time was the main constraint and one third blamed the setting and environment.

The experience of the OSBE portrays a new learning method, as it was applied for formative assessment of undergraduates as a pilot project for our course in biostatistics. Nevertheless, it was a new learning experience not only for the students but also for the faculty members. Of course, it had certain limitations, such as the fact that each station was designed to be completed in 2 minutes. We did have two reasons for the 2 minute limit per item: first, it was a pilot study, and second, according to our tests, two minutes was enough time to perform required commands; however, it was not equal to other examinations that usually give 10 to 15 minutes per item. Thus, it is difficult to compare the OSBE with other related examinations [6, 10, 11].

Since both phases (Year 2009, 2010) are not similar, so we tested different commands for each year, and the two phases were scored differently and in phase 1 examined groups in reverse order (G4 to G1). However, we have analyzed the association of scores between genders and among different groups. The results depict that the mean score of the females in the Phase 1 examination was higher than that of the males (P<0.05), but there was no significant difference between the males and females in phase 2. In view of the fact that in the last a few decades, the role of gender in learning process has drawn attention and debate [12, 13], it is worth considering what could account for the small but significant gender difference that we observed in our study. The answer could have simply been that the individual females in phase 1 took the test more seriously and worked hard to prepare. We need to further explore the reasons in future studies. There are significant (P<0.05) differences in the mean score in phase 1 & 2 among the four groups. We made our best attempt to prevent each group of students from contacting the other students who were waiting for their exam, which is necessary to reduce the bias in results. However, we cannot be completely certain that none of the students communicated with each other; therefore, this might be a justification of the difference in scores among the four groups and also shows a limitation of our study. When we compared the two parallel groups A & B, there is a slight difference present in the mean scores of both groups in phase 1, whereas there is no significant difference present in the groups of phase 2.

As it is a part of formative assessment, brief feedback was given for the purpose of learning and improvement. After analyzing the results, there was a group discussion among the students and examiners. The majority of students were satisfied with the process and appreciated that they also learned or practiced how to use the computer for data analysis. However, almost all reported that they were stressed by the exam and a few of them felt that the time provided was not appropriate; on the other hand, almost one third of the students agreed that it was a simple and quick examination. They even believed that it had more objectivity than other assessment tools, yet the students emphasized that they wanted to have more training.

Certainly, the matter of validity and reliability is important for any assessment tool. Though this pilot study project shows an inter-rater reliability level (0.60) that was not very high, it was still acceptable. We believe that this issue can be easily resolved by examining more students at a same time by increasing stations and groups and perhaps by randomly re-checking [14]. Since the examination was completed in a half day with almost 200 students, tried to conduct it as possible as objective saved the cost of paper, and required less effort for checking and scoring; therefore, it seems that it is a practical and feasible examination process. We believe that there were additional learning advantages that occurred in the students who participated in this method of assessment:

1. The students were prepared for further assessment in a more stressful condition with appropriate time management.
2. The students were sensitized to the technical aspects of computer skills and managed to handle data in a practical way and understand the analytical approach that is required for the understanding of application of statistics in health care.

In conclusion, our findings suggest that we can use a computer easily and effectively in formative examination of biostatics. However, it requires further planning and training in order to maintain objectivity and not to have biased results. Confirmatory studies are still required to support our conclusion on a large scale.

## Figures and Tables

**Table 1:**
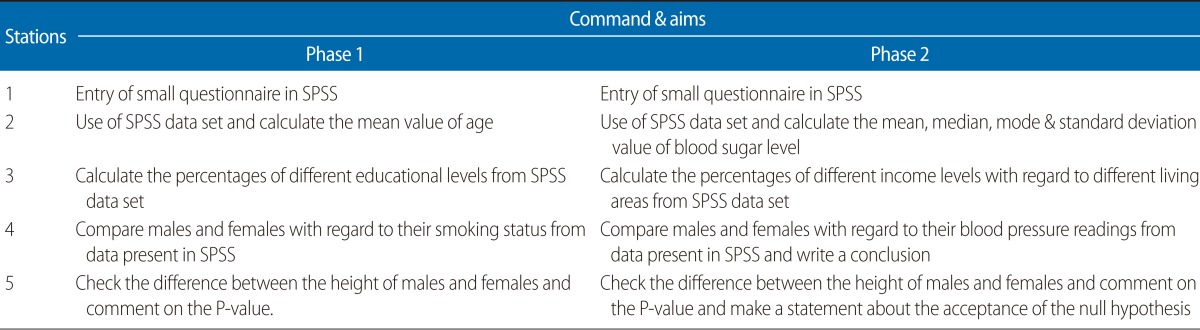
Five stations of the objective structured biostatistics examination for phase 1 and 2 students

**Table 2:**
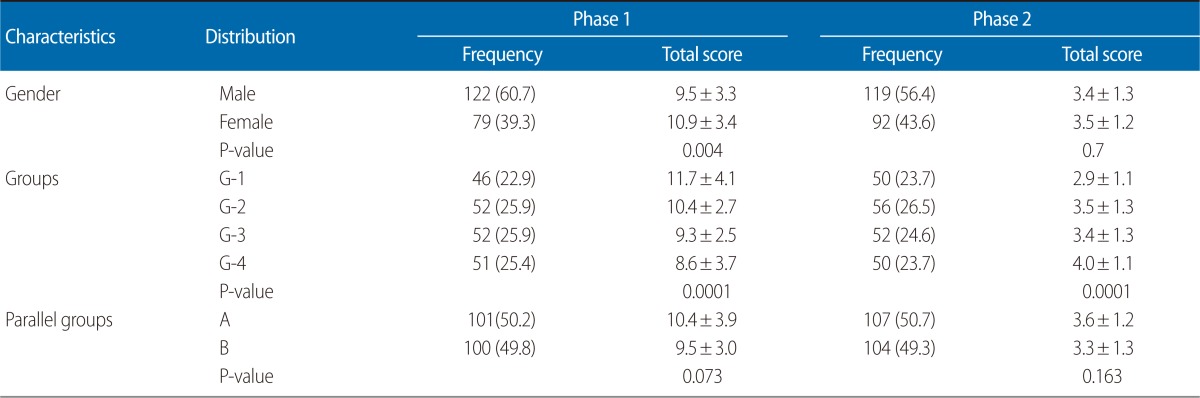
Results of the objective structured biostatistics examination for phase 1 and 2 students

Values are presented as number (%) or mean±SD.
